# Influence of Cutting Regime Parameters on Determining the Main Cutting Resistance during Polypropylene Machining

**DOI:** 10.3390/polym16111537

**Published:** 2024-05-29

**Authors:** Slavica Prvulović, Predrag Mošorinski, Ljubiša Josimović, Jasna Tolmač, Luka Djordjević, Mića Djurdjev, Mihalj Bakator, Branislava Radišić, Dejan Bajić

**Affiliations:** 1 Technical Faculty “Mihajlo Pupin”, University of Novi Sad, 23000 Zrenjanin, Serbia; slavica.prvulovic@tfzr.rs (S.P.); jasna.tolmac@tfzr.rs (J.T.); mica.djurdjev@tfzr.rs (M.D.); mihalj.bakator@tfzr.rs (M.B.);; 2 College of Textile Engineering, Textile High School of Professional Studies, 16000 Leskovac, Serbia; ljubisa.josimovic@vsstle.edu.rs

**Keywords:** machining, cutting resistance, polypropylene, PLC, CNC lathe, manufacturing efficiency

## Abstract

This study examines the impact of cutting regimes on determining cutting resistance in the processing of polypropylene (PP) using the CNC lathe EMCO F5. The rationale for this research stems from polypropylene’s rarity among thermoplastics in possessing structural stability, allowing for its comparison to metals and practical application in products replacing metal parts. Leveraging its favorable mechanical properties, polypropylene finds utility in producing parts subject to dynamic loads, boasting high resistance to impact loads—particularly undesirable in machining. An advantageous characteristic of polypropylene is its affordability, rendering it an economical choice across numerous applications. Despite these merits, polypropylene’s exploration in cutting processing remains limited, underscoring the novelty of this research endeavor. The main method for determining cutting resistance involves measuring electric current strength during processing. This direct measurement, facilitated by input cutting regime parameters, is recorded by the PLC controller, with the current value extracted from the machine tool’s ammeter. The experimental approach entails varying cutting regime parameters—cutting speed (v), feed rate (s), and depth of cut (a)—across minimum and maximum values, recognized as pivotal factors influencing cutting force development and the attainment of the desired machined surface quality.

## 1. Introduction

Thermoplastic materials have emerged as highly advantageous for prototyping parts and devices across diverse developmental stages. Their exceptional mechanical, thermal, frictional, and electrical properties make them indispensable in modern industrial products, spanning automobiles, machine tools, household appliances, electric machinery, agricultural implements, food processing equipment, chemical apparatus, construction machinery, and more. Their versatility extends to a wide array of components and assemblies, including gears, sprockets, sliding bearings, sleeves, levers, seals, sliding tracks, insulators, pipelines, containers, and housings. Moreover, they excel in the manufacture of models with larger dimensions, where production costs are critical [1,2,3,4].

The extensive utilization of thermoplastic materials is primarily attributed to their recyclability, cost-effectiveness, ease of machining with low cutting forces and temperatures, resilience to external factors, and numerous other desirable properties [5,6]. Operating within various temperature ranges specific to each thermoplastic ensures the prevention of excessive heating and the preservation of mechanical and other essential properties [1,7,8,9,10,11]. Ongoing advancements in thermoplastic materials, often termed engineering plastics, are directed towards enhancing mechanical characteristics, refining chemical compositions, and developing composites that better align with market demands.

Machining plastic materials poses unique challenges that markedly differ from metal machining. Plastic machining entails excellent insulation properties, resulting in significantly reduced heat transfer across the cross-sectional area compared to metals [1,11,12,13]. Despite their advantageous insulating characteristics, plastic materials inherently possess inferior mechanical properties. Consequently, lower temperatures are expected to develop in the cutting zones of the workpiece and tool, primarily attributed to the low friction coefficient at the contact surface between the tool and the workpiece [1,14,15,16,17]. However, challenges arise if elevated temperatures occur on the surface layer during the cutting process. Controlled temperature management becomes essential through the selection of cutting regime parameters and optimization of the cutting process to ensure that workpiece temperatures remain within optimal limits for specific thermoplastics [18,19,20,21].

Due to their generally inferior mechanical properties compared to metals, thermoplastics are often preferred for processes requiring more intensive cutting regimes [20,21,22]. One of the most advantageous properties of thermoplastics is their low thermal conductivity, which allows for intense heating of the surface layer while minimizing effects on the interior [23,24]. While this property is desirable for machining by cutting, it necessitates the implementation of effective surface-layer-cooling methods to prevent disruption of the base material’s chemical structure during cooling and ensure the workpiece reaches an appropriate temperature. However, excessive heating of the surface layer can lead to significant dimensional distortions in the workpiece, surpassing specified tolerance levels due to a high coefficient of thermal expansion. Consequently, machining thermoplastic materials is inherently unpredictable and relatively more complex compared to machining metals [1,25,26,27].

The primary machining processes for removing chips from thermoplastic materials include turning, milling, and drilling, integral to the overall product-shaping process [28,29,30]. However, in modern environments, the practice of polishing thermoplastic products is experiencing a notable rise in adoption. Initially pursued for aesthetic enhancements, polishing is now increasingly applied for specific functional purposes, particularly in fields such as medicine, the chemical industry, automotive manufacturing, and others [31,32,33]. While advanced technology machining processes like water jet machining, laser machining, and chemical machining have emerged, traditional chip removal machining processes continue to dominate the industry [2,7,8,11].

The principal drawback of thermoplastics lies in their lack of structural stability, a characteristic that sets them apart from metals and largely defines their utility [34,35]. Among thermoplastics, polypropylene stands out as a rare example possessing significant structural stability, comparable to metals in this regard [36,37]. This quality enables its practical application in manufacturing products that substitute metal parts. Leveraging its commendable mechanical properties, polypropylene finds extensive use in producing parts exposed to dynamic loads. Furthermore, its inherent hardness imparts remarkable resistance to impact loads, which are particularly undesirable in machining processes [38,39,40,41].

Polypropylene is renowned as one of the lightest plastics, boasting a density of approximately 0.92 g/cm^3^. In its natural form, it exhibits a colorless and odorless profile, and crucially, it is non-toxic and safe for human use. These attributes, coupled with its versatile physical and chemical properties, render it indispensable across industries and in everyday life [38,39,42]. Polypropylene finds wide-ranging applications in various sectors, including pharmaceutical packaging, where its high chemical resistance makes it ideal for storing aggressive substances. Additionally, it is extensively employed in medical equipment such as syringes and medicine packaging [43,44]. Furthermore, its commendable mechanical and thermal properties position it as a preferred construction material for machine components, covers, and housings across diverse industries. Its favorable electrical properties further augment its utility, making it a staple in the electronics industry for producing various equipment and apparatus elements.

Polypropylene stands out for its resistance to moisture absorption and corrosion, making it well-suited for environments exposed to humidity or harsh atmospheric conditions [45,46]. Its combination of lightweight and strength makes it particularly desirable in applications where material strength is crucial without adding unnecessary weight. Moreover, its ease of processing and shaping provides flexibility in product design, enhancing its usability across various industries. A significant advantage of polypropylene lies in its affordability, rendering it an economical choice for a wide array of applications [47,48,49].

Polypropylene is frequently selected for its remarkable ability to withstand a variety of harsh conditions, including fluctuating weather patterns, chemical aggressiveness, and rigorous mechanical demands. This durability renders it indispensable across numerous industries, serving a wide range of applications. In both everyday life and business settings, polypropylene has become a staple due to its exceptional resilience, chemical resistance, ease of processing, and, notably, its affordability [40,41,42,50].

During the machining of thermoplastics, it is common to set the back rake angle of the cutting tool (α) within the range of 5° to 10°, while the front rake angle (γ) varies depending on the material being processed. For polypropylene, the front rake angle typically ranges from 0° to 5°, while for Teflon processing, the angle is between 5° and 8°. The knife angle (κ) for machining polypropylene usually ranges from 45° to 60°, while for Teflon, it is around 10°. Cutting speeds (v_c_) typically range from 250 to 500 m/min for polypropylene, and from 150 to 500 m/min for Teflon. The feed rate (s) for polypropylene plastic falls within the range of 0.1 to 0.457 mm/rev, whereas for PTFE plastic, it typically ranges from 0.1 to 0.30 mm/rev [51].

To ensure the uniform quality of the machined surface, it is essential to reduce the radius of the tool tip. A smaller radius mitigates vibrations and guarantees a more consistent and accurate machining process [52,53].

In the evolving landscape of material engineering, the machining of thermoplastics has garnered considerable interest due to their versatile applications in diverse industries. Among these materials, polypropylene is particularly noteworthy for its mechanical properties and cost-effectiveness, making it a viable substitute for metals in numerous engineering applications. Despite its widespread use, detailed studies focusing on the machining characteristics of polypropylene are sparse in the existing literature, particularly concerning CNC lathe operations.

This study addresses this gap by exploring the influence of various cutting regime parameters on the main cutting resistance during the CNC machining of polypropylene. Leveraging advanced measurement techniques and the analytical capabilities of a Programmable Logic Controller (PLC), this research provides a meticulous examination of how different machining parameters—cutting speed, feed rate, and depth of cut—affect the cutting resistance. The insights gained from this study are poised to refine the machining process for polypropylene, enhance operational efficiencies, and reduce the ecological footprint of production practices, aligning with the ongoing shift towards sustainable manufacturing processes.

By integrating a methodologically rigorous approach with practical industrial applications, this research not only contributes to the academic discourse but also serves as a valuable resource for engineers and manufacturers seeking to optimize machining processes for polypropylene and similar thermoplastic materials.

The novelty of this study is manifold and significant, particularly in its exploration of polypropylene machining—a subject that has seen limited examination in the realm of CNC turning operations. By focusing its analysis on this material, the research fills a critical gap in the existing scholarship and significantly enriches our understanding of its unique machinability characteristics. This advance is not merely academic but has profound implications for industrial applications where polypropylene is used.

Furthermore, the research framework employed here is meticulously designed to investigate the impact of various cutting parameters on the main cutting resistance encountered during machining. This comprehensive experimental approach not only sheds light on the optimal conditions for machining polypropylene but also provides deep insights into the complex dynamics of the machining process itself. This study methodically dissects these dynamics, offering a clear view into how specific adjustments in cutting parameters can lead to improvements in both efficiency and product quality.

In doing so, this research not only navigates uncharted territories in polypropylene machining but also sets a new benchmark for future studies. It lays a robust foundation for subsequent inquiries and technological advancements, ensuring that the findings here can be leveraged to enhance the precision and sustainability of manufacturing practices involving thermoplastics. By establishing these critical connections between academic research and practical applications, this study underscores the growing importance of adaptability and innovation in modern manufacturing landscapes.

Moreover, this study integrates sophisticated measurement techniques, including PLC-controlled data acquisition and analysis, thereby augmenting the precision and dependability of the experimental findings. This methodological rigor underscores a steadfast commitment to scholarly exactitude and meticulousness. In sum, the amalgamation of a pioneering research focus, stringent experimental methodology, and the incorporation of advanced technological modalities distinguishes this study and elevates its standing as a noteworthy contribution to the domain of machining and materials processing.

## 2. Materials and Methods

### 2.1. Influence of Force on the Cutting System

The cutting force plays a pivotal role in machining operations, carrying multifaceted significance. Together with cutting speed, it determines the power required by the drive motor. Additionally, it interacts with the force of penetration resistance, assessing the durability of the tool holder, and serves as a crucial parameter in computational assessments of a material’s workability. In turning operations, the main cutting force contributes significantly, accounting for 70% to 80% of the total cutting force—making it the predominant component. Consequently, analyzing the cost-effectiveness and conditions of turning machining often focuses solely on this component [25,52,54,55].

Accurately determining the main cutting force holds paramount importance across various aspects of machining operations. It facilitates precise machine power calculation, informs proper tool design, aids in selecting optimal cutting parameters, guides the choice of appropriate workpiece support and clamping methods, enables real-time monitoring and adjustment of the machining process during operation, and ensures process stability with minimal vibrations. Moreover, it contributes to enhancing the cost-effectiveness of the production process [25,54].

In recent years, analytical or numerical methods have gained prominence for predicting cutting forces, streamlining production design processes to achieve more economically viable outcomes compared to experimentally generated production parameters. Process modeling primarily focuses on orthogonal cutting to enhance understanding, while three-dimensional (oblique) cutting scenarios are often simplified and projected onto orthogonal cutting for easier comprehension of the more complex oblique cutting processes [25,54].

**Figure 1:**
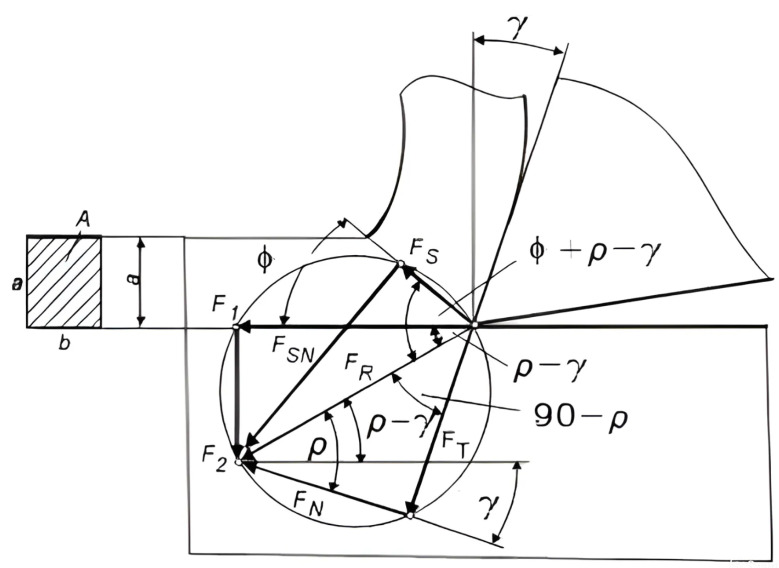
Cutting force (F_R_) in orthogonal cutting. Where: F_R_—cutting force; F_1_—main cutting force resistance; F_2_—penetration resistance.

Figure 1 depicts forces in orthogonal cutting.

The analysis of chip formation while cutting hinges on the defined shear angle (Φ) is a crucial parameter for understanding the process. This angle primarily governs the plastic deformation of the chip and subsequently influences its separation from the base material. The cutting action generates shear force (F_s_) and normal shear force (F_sn_) collectively contributing to the cutting force (F_r_). The shear angle (Φ) holds multifaceted significance and serves as a key criterion for assessing material machinability. Increasing this angle typically leads to improved machined surface quality, a reduction in cutting force components (F_1_, F_2_, F_3_), and the production of ribbon-like chip shapes—a desirable outcome when machining thermoplastic materials.

### 2.2. Technical Preparation of the Experiment

The measurement of the main cutting resistance (F_1_) was conducted in the machine laboratory using a sample of a specific workpiece made of polypropylene (PP). This material has become indispensable in both everyday life and business due to its exceptional durability, chemical resistance, ease of processing, and affordability. With excellent prospects for the future and favorable operational characteristics [38,56,57,58], polypropylene is highly regarded in various applications. Considerations regarding the influence of static and dynamic factors on the process flow, machining accuracy, and other impacts on cutting force guided the selection of a machine with a precision of 0.01 mm.

The chosen machine for the experiment was the EMCO CNC F5 lathe, selected for its several comparative advantages, despite having some limitations such as a restricted range of feed speeds (v_f_) and workpiece diameter, among others. The core basic components for conducting the described experiment include the workpiece made of polypropylene (PP), the machine used for processing (in this case, a CNC lathe (EMCO CNC F5)), the cutting tool, and the computerized measuring system integrated with accompanying equipment, where measurements have been performed.

The experimental method employed in this study entails varying cutting parameters, including cutting speed (v), feed rate (s), and cutting depth (a), across their minimum and maximum values. These parameters exert a significant influence on the development of cutting force, thereby playing a crucial role in attaining the desired quality of processed surfaces. Furthermore, the current intensity is monitored on the ammeter of the selected machine to calculate variations in electric current intensity exhibited during the experiment (k_s_), which is utilized for scaling the PLC.

The measurement of the main cutting resistance is conducted using a thermocouple connected to the controller, facilitating the transmission of the detected signal to the computer for processing. The acquired data will be presented in both tabular and graphical formats to facilitate easier analysis and enhance clarity.

To establish a comprehensive database of cutting parameters and corresponding values of the main cutting resistance, an experiment is conducted on a material with a nominal diameter of Ø40 mm and a length of L = 400 mm. The cutting tool utilized is equipped with a hard metal insert (DIN 4976 1010 P10) featuring a cutting edge angle of k = 45°. For workpiece support and stability during the experiment, a collet chuck and a tailstock center are employed as the workpiece holder, ensuring the necessary stability throughout the process. Efforts were made to minimize the influence of surrounding machines on the machining process.

**Figure 2:**
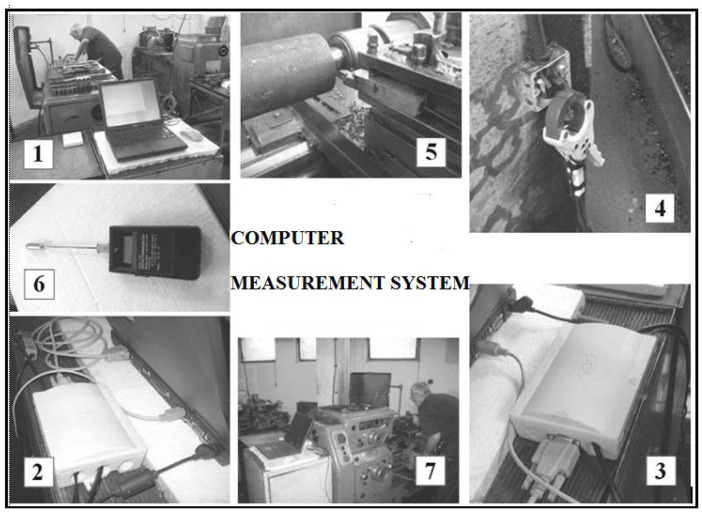
Computerized measurement system for detecting cutting force. Picture 1 and 7—photographs of the computer and the universal lathe equipped with a turner, Picture 2 and 3—the programmable logic controller, Picture 4—the measuring clamps, Picture 5—the tool holder with the turning tool, Picture 6—auxiliary temperature gauge.

The computerized measurement system for measuring the cutting force of machining plastics is illustrated in Figure 2. Pictures 1 and 7 display photographs of the computer and the universal lathe equipped with a turner. Pictures 2 and 3 showcase the programmable logic controller (PLC). Picture 4 presents the measuring clamps gripping a single phase of the motor stator. In picture 6, an auxiliary temperature gauge of the surrounding air is depicted. Finally, picture 5 depicts the tool holder with the turning tool.

**Figure 3:**
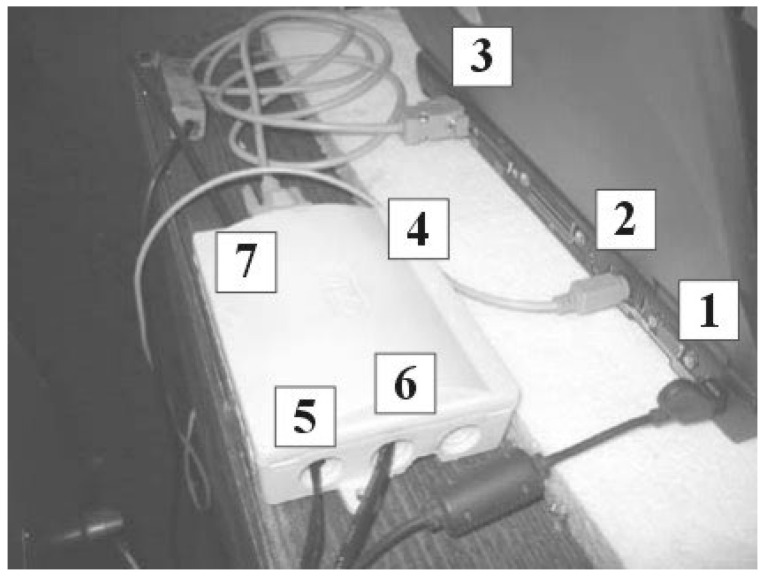
Computer power supply and data transmission system.

In Figure 3, the power supply of the computer (1) is depicted along with the mouse connector (2) and the RS232C connector (3). The PLC (4) is powered by an AC/AC adapter (5), with the current clamp connector (6) and the temperature sensor connector (7).

The PLC’s primary task is to convert the collected data, represented by numerical values ranging from 0 to 5 volts from the temperature sensor and current clamp, into binary code. These binary data are then transmitted to the computer for analysis and processing.

Current clamps are utilized to measure the current flowing through a supply cable without the need to interrupt the circuit. They typically consist of coils wound around a toroidal ferrite core, which facilitates the induction of lines of magnetic force. The transfer ratio of current clamps is 1/1000 [A].

### 2.3. Workpiece

**Figure 4:**
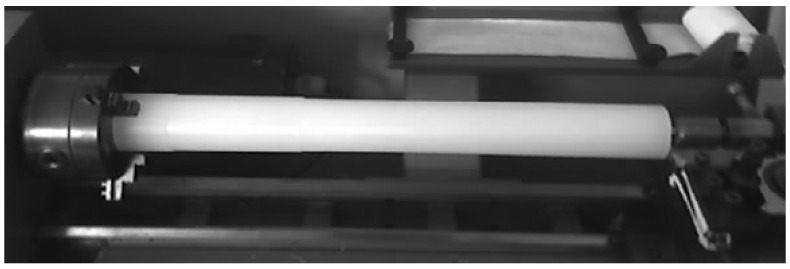
Workpiece.

Figure 4 shows the workpiece made of polypropylene of dimensions Ø40 × 400 mm. The workpiece holder consists of a chuck and a tailstock center, facilitating its revolving motion during machining.

### 2.4. Force Measurement in the Cutting Zone

The measurement of the cutting force involves generating electrical current strength, which varies under specific conditions and is dependent on cutting parameters, while assuming a constant voltage throughout the experiment. The PLC controller directly records the electrical current intensity, facilitated by the input parameters of the cutting regime. Concurrently, the current value of the force is monitored on the ammeter of the machine tool. This dual detection of electrical current intensity enables the calculation of a corrective coefficient, which is then used for filtering the measured values. The specific amount of force is generated through a mathematical method based on a provided formula, calculated using measurable parameters determined in the equation.

## 3. Results and Discussion

### 3.1. Generating Main Cutting Resistance on a EMCO F5 CNC Lathe

The principal methodology employed for data acquisition in this study is experimental in nature. Data are gathered through the execution of experiments on manufacturing machinery, employing specialized measurement instruments, and feeding the collected data into the computer system through the Programmable Logic Controller (PLC) interface. Following this acquisition phase, the gathered data undergo processing procedures aimed at enhancing clarity and facilitating purposeful representation across a range of formats, encompassing tabular, graphical, and integrated formats.

Another method utilized is the indirect method, which involves employing mathematical models to determine results when direct measurement of relevant quantities is not feasible. This method also serves to verify the experimentally obtained results.

Considering that the experiment is conducted within real-time production environments, akin to laboratory settings mimicking actual production conditions, it is plausible for certain discrepancies in measurements to arise owing to the impact of the immediate surroundings on the machinery utilized in the experiment. Furthermore, fluctuations in electrical current parameters such as voltage and current intensity are foreseeable, thereby validating the adoption of diverse data generation models to derive cohesive conclusions.

The primary cutting resistance during turning (F_1_) is directly influenced by the cutting depth (a) and the feed rate (s), as well as coefficients specific to the material being processed. Other components that affect the cutting resistance force in the turning process are determined in relation to the main cutting resistance (F_1_:F_2_:F_3_ = 5:2:1) [27,38,39]. However, these components are not the focus of consideration in this study.

Generating the main cutting resistance can be achieved through direct measurement using a dynamometer, followed by transforming input impulses via the computer’s input port based on cutting regime parameters defined and registered by the controller. Alternatively, another method involves measuring electrical components and obtaining the desired values through a mathematical approach. Given that the latter method is more cost-effective and straightforward, albeit less prevalent in practice, force generation will be conducted in this manner. The CNC machine, with its technical characteristics, facilitates direct readings of electrical components, primarily current intensity, registered on the controller. Subsequently, scaling is used to obtain the actual value, which is then used to define the force using Equation (1):

(1) $$F_{1}=\frac{U\cdot I}{L\cdot \omega },$$

where the values are as follows:

U—voltage (constant value of 220 V);

I—current intensity (A);

L—free length of the tool holder handle (L = 0.020 m);

ω—angular velocity (rev/s) of the workpiece.

The assumed equipment used for conducting the experiments allows for both methods of generating main cutting resistance. However, the analysis will be primarily conducted on a CNC machine due to its high movement precision, stability in the machining process, and the potential application of artificial intelligence (AI) systems for machining, such as fuzzy logic.

The initial conditions for generating the main cutting resistance are provided in Table 1.

Where:

a_p_ (mm)—depth of cut;

n (rpm)—rotational speed;

v_f_ (mm/min)—cutting speed.

The measurement method involves conducting eight measurements by varying the given variable cutting regime parameters.

**Figure 5:**
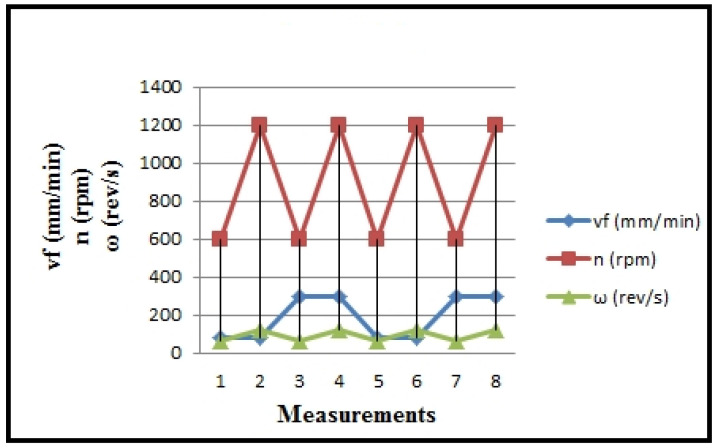
Values of calculated cutting regime parameters v_f,_ n, ω.

**Figure 6:**
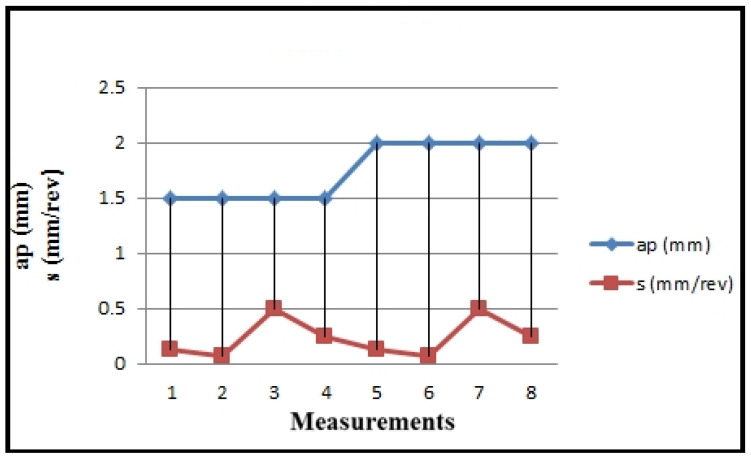
Values of calculated cutting regime parameters a_p_, s.

The overview of the input variables modified on turning technology (feed rate—s) is presented in Table 2, along with the angular velocity parameter (ω), and in Figure 5 and Figure 6. These values are calculated based on the specific technological characteristics of the machine.

(2) $$v_{f}=s\cdot n\;[mm/min],$$

Based on which we obtain the following:

(3) $$s=\frac{v_{f}}{n}[mm/rev],$$

In contrast, angular velocity can be obtained according to the following equation:

(4) $$ɷ=\frac{\pi \cdot n}{30}[rev/s]$$

### 3.2. Measuring Elements

The number of revolutions (n) of the workpiece, i.e., the machined part, is measured by a tachometer equipped with a potentiometer, providing a range from 16 to 100% on the measurement scale. The minimum value on the scale is n_min_ = 16. The desired revolution value is read on the display of the control panel of the CNC lathe EMCO F5 CNC. Revolutions range between 50 and 3000 rpm, with a maximum value of n_max_ = 100, resulting in a range R = n_max_ − n_min_ = 84%.

There are a total of six groups of revolutions. The electric current of the motor, I, is measured by an ammeter. The minimum value on the scale is I_min_ = 0(A), while the maximum value is I_max_ = 5(A), resulting in a range R = I_max_ − I_min_ = 5(A). There is a total of 5 coarse (1 A), 10 medium divisions (0.5 A), and 50 fine divisions (0.1 A). The sensitivity and accuracy are equal, and amount to 0.1 A. The measured value can be read on the display of the CNC lathe control panel. The speed of feed, v_s_, of the workpiece part is measured by a speedometer equipped with a potentiometer, providing a range from 5 to 400 mm/min on the measurement scale. The minimum value on the scale is v_smin_ = 5  (mm/min), while the maximum value is v_smax_ = 400  (mm/min), resulting in a R =  v_smax_ − v_smin_ = 395 (mm/min). There is a total of six divisions, and the sensitivity and accuracy are not cataloged but are based on empirical experience. The measured value can be read on the display of the CNC lathe control panel.

### 3.3. Processing and Presentation of Measurement Results

**Figure 7:**
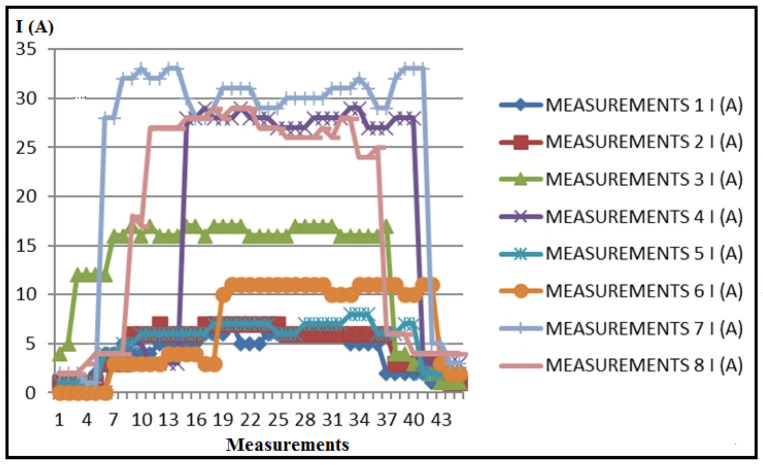
Electric current measurement results, I (A).

Based on the tabular results, which are not presented in this document due to volume, the processed results of the measured electrical current of the stator of the electromotor in the numerical lathe, I (A), obtained through PLC measurements, are presented in Figure 7.

**Figure 8:**
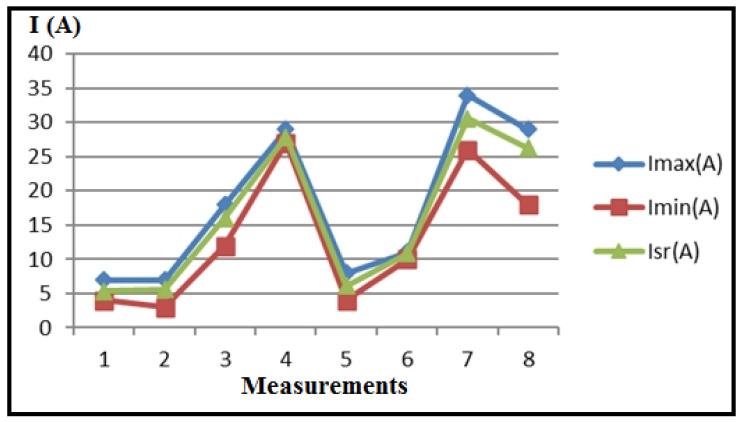
Statistics of the measured current values (I).

**Figure 9:**
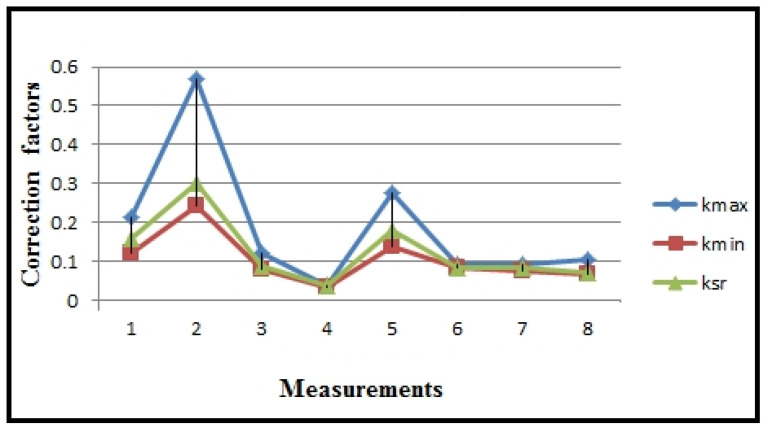
Statistics of the correction factor results.

Table 3 and Figure 8 present the measured values of electrical current, recorded by the ammeter of the CNC lathe, along with the statistics (I_max_, I_min_, I_sr_) of numerous sequences for eight measurements during plastic turning under different regimes. Correction factors for scaling the PLC (k_max_, k_min_, k_sr_) are calculated and included in Table 3 and Figure 9, using the formula k_max,min,sr_ = I_A_/(I_PLC_)_max,min,sr_.

Analysis of the maximum current values obtained reveals that measurements 7 and 8 recorded the highest values. The cutting regimes for these measurements are as follows: measurement 7 (a_p_ = 2 mm; v_f_ = 300 mm/min; n = 600 min^−1^); measurement 8 (a_p_ = 2 mm; v_f_ = 300 mm/min; n = 1200 min^−1^). Given that the cutting depth (a_p_) and feed rate (v_f_) are set to their maximum values for this experiment, it can be concluded that these two parameters have the most significant impact on the variations observed during machining.

The minimal electric current generation is evident in measurements 1 and 2, with cutting regime parameters as follows: measurement 1 (a_p_ = 1.5 mm; v_f_ = 80 mm/min; n = 600 min^−1^), and measurement 2 (a_p_ = 1.5 mm; v_f_ = 80 mm/min; n = 1200 min^−1^). This also confirms the previous statements.

Table 4 provides experimental data on current variations during the measurement process.

**Figure 10:**
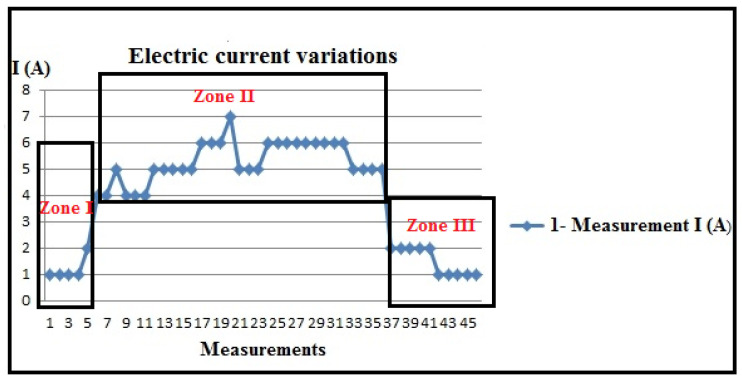
Current diagram for measurement number 1.

Based on the tabular and diagrammatic representation, characteristic periods of current detection (I) are observed during processing (Table 4 and Figure 10).

1. Current detection zone during tool positioning without processing;
2. Current detection zone during processing;
3. Current detection zone as the tool exits the engagement.

These characteristic zones are distinctive as follows:

Zone 1: The zone of tool approach to the workpiece detects a certain current intensity in most cases, indicating that the drive motors are loaded due to the resistance to movement caused by friction. Friction occurs between coupled elements in the transmission for main and auxiliary movements, in the bearings of drive motors, on sliding surfaces for certain directions of movement (such as movements along coordinate axes), and similar areas. The beginning of the cutting processing, i.e., the entry of the tool into engagement, is also crucial. The diagram clearly shows the slope of the current intensity detection line, indicating the gradual and uniform loading at the beginning of the cutting process. This is significant for energy consumption and the possibility of vibrations at the beginning of the processing. The steeper the slope of the curve, the smaller these parameters, as observed in measurements 1, 2, 5, and 6.

In the course of these measurements, variations occur in cutting depths and cutting speeds, whereas the steps (feeds) remain consistent. As a result, it can be deduced that the feed rate stands out as the most significant parameter in non-cutting processing, emphasizing the criticality of the tool’s approach speed for energy conservation in this context.

**Figure 11:**
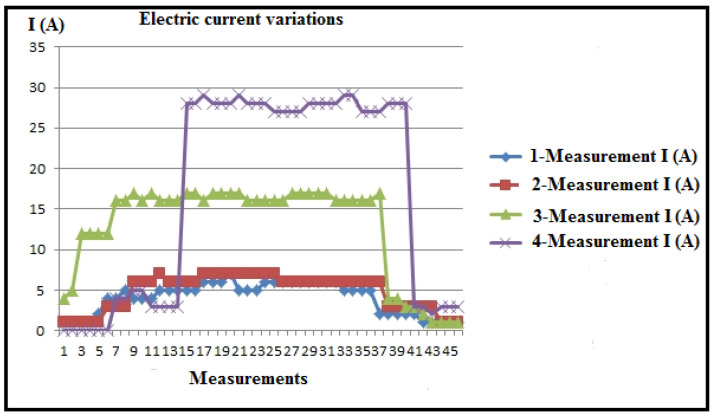
Electric current diagram for a_p_ = 1.5 mm.

**Figure 12:**
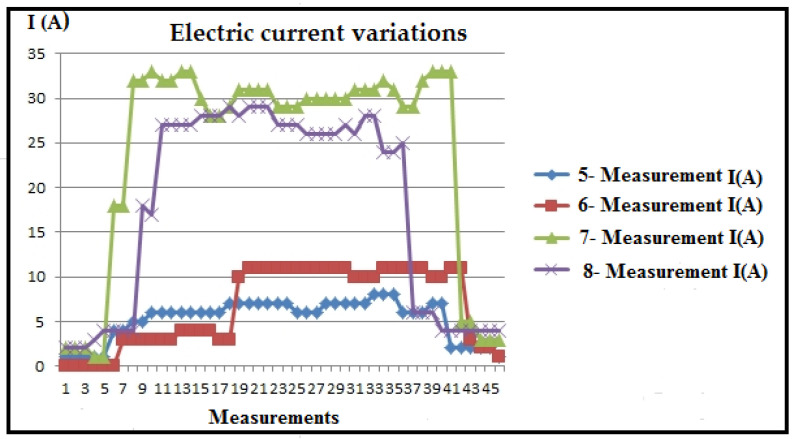
Electric current diagram for a_p_ = 2 mm.

Zone 2: The second zone, known as the immediate processing zone, is characterized by prominent current peaks at certain moments (Figure 11 and Figure 12). This phenomenon is attributed to material non-homogeneity, increased friction when the chip slides on the rake face of the tool, radial deviation during material rotation, and friction on sliding surfaces of the tool. These deviations in current are not fundamentally an overload of the system but are visible depending on whether the processed surface is untreated (outer layer) or partially and completely processed in the initial stages. Given the very low coefficient of sliding for thermoplastic materials, in general, current variations are very low and almost negligible.

During the machining process, an increase in friction was observed, attributed to the maximum cutting depths reached and partial blade wear resulting from previous tool operations. It can be hypothesized that during these measurements, the machine’s sliding surfaces partially dried, causing heightened friction. Eventually, insufficient lubrication of these surfaces resulted in intermittent movement, a phenomenon known as “stick-slip” motion. At certain junctures during the measurements, the recorded results were deemed unacceptable, prompting the exclusion of those data points, as elucidated in specific studies, offering detailed explanations and justifications. The diagram provided illustrates the repetition of measurements. The overarching conclusion underscores the imperative need for consistent lubrication of the machine’s sliding surfaces, especially when processing longer workpieces, presenting a notable challenge.

Zone 3: The third zone corresponds to when the tool exits the cut and exhibits similar characteristics to the tool entry zone (Zone 1). The main takeaway is that adopting the oblique cutting principle proves much more effective than orthogonal cutting in reducing (and neutralizing) vibrations during both the entry and exit of the lathe tool.

**Figure 13:**
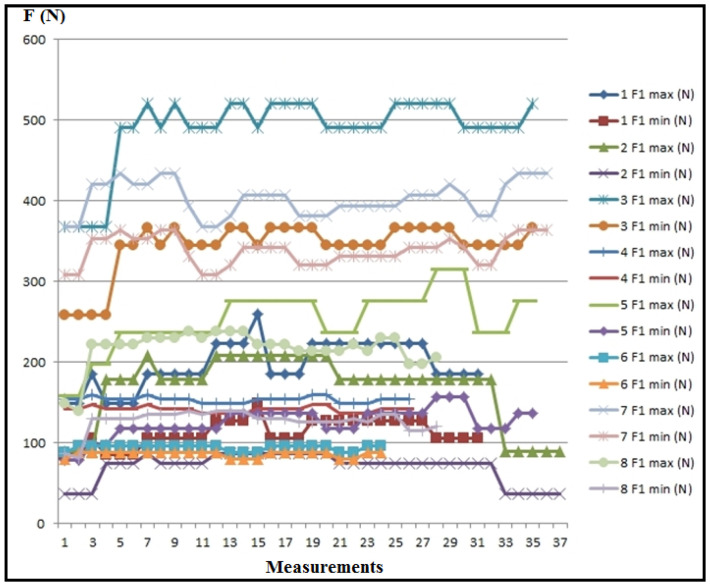
Values of the main cutting resistance for individual experimental measurements.

**Figure 14:**
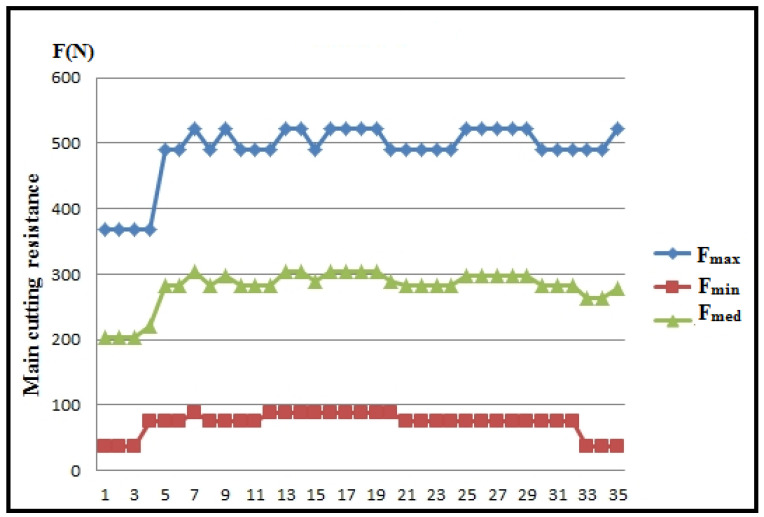
Maximum, minimum, and mean values for the main cutting resistance.

Figure 13 showcases the values of the main cutting resistance for each experimental measurement, while Figure 14 depicts the maximum, minimum, and mean values of the main cutting resistance.

Based on the diagrams (Figure 13 and Figure 14), it is evident that the main cutting resistance reaches its peak in measurement 3 (F_1max_), where a_p_ = 1.5 mm, v_f_ = 300 mm/min, s = 0.5 mm/rev, n = 600 rpm, ω = 62.8 rev/s. Conversely, the lowest values of the main cutting resistance are observed in measurement 2 (F_1min_), where a_p_ = 1.5 mm, v_f_ = 80 mm/min, s = 0.067 mm/rev, n = 1200 rpm, ω = 125.6 rev/s. This suggests that the feed rate significantly influences the main cutting resistance. As the feed rate increases, the cutting resistance also rises, while an increase in speed tends to reduce the cutting resistance, provided other parameters remain constant.

## 4. Conclusions

The turning experiment conducted on polypropylene (PP) material, with a nominal diameter of Ø40 mm and a length of L = 400 mm, aimed to establish a comprehensive database of cutting regimes and determine the values of the main cutting resistance.

The measurement of the cutting force involved generating electrical components that varied under specific conditions, depending on cutting parameters, and assuming constant voltage. Direct measurement of electric current strength was facilitated by the input cutting regime parameters, recorded by the PLC controller. Meanwhile, the current value was read from the ammeter of the machine tool.

The processed measurement results are depicted in tables and diagrams, providing insights into the values of the main cutting resistance for each experimental measurement, visually represented.

Characteristic periods of current strength detection (I) during processing are identified, leading to the determination of the main cutting resistance values in different zones: during tool positioning without processing, during processing, and during tool exit from engagement.

These studies mark a breakthrough in the processing of polypropylene, as this material has been relatively unexplored in terms of cutting processing, particularly in the context of CNC lathe operations. Based on all the foregoing, we conclude that this study makes a valuable contribution to understanding the process of machining polypropylene on CNC lathes.

Further research should delve into a more granular analysis of individual processing parameters. There is considerable scope for developing advanced machining techniques that could revolutionize the production of polypropylene components, making the process more efficient and cost-effective. Additionally, integrating cutting-edge technologies such as artificial intelligence, with applications of fuzzy logic or neural networks, promises to enhance parameter optimization and improve overall machining precision.

This study serves as a foundational contribution to the field, offering a platform for future investigations into the machinability of polypropylene and other thermoplastic materials. It paves the way for exploring sustainable machining practices that align with the principles of green manufacturing and resource efficiency, which are becoming increasingly crucial in the global push towards sustainable industrial practices.

## Figures and Tables

**Table 1:** Values of input cutting regime parameters.

	Min	Max
a_p_ (mm)	1.5	2
v_f_ (mm/min)	80	300
n (rpm)	600	1200

**Table 2:** Values of calculated cutting regime parameters.

		Measurements							
		1	2	3	4	5	6	7	8
a_p_	(mm)	1.5	1.5	1.5	1.5	2	2	2	2
vf	(mm/min)	80	80	300	300	80	80	300	300
s	(mm/rev)	0.133	0.067	0.500	0.250	0.133	0.067	0.500	0.250
n	(rpm)	600	1200	600	1200	600	1200	600	1200
ω	(rev/s)	62.8	125.6	62.8	125.6	62.8	125.6	62.8	125.6

**Table 3:** Statistics of the measured current values (I) and correction factor for ammeter/PLC.

		Measurements							
		1	2	3	4	5	6	7	8
Ammeter	A	0.85	1.7	1.4	1	1.1	0.9	2.5	1.9
PLC									
I_max_	A	7	7	18	29	8	11	34	29
k_max_		0.2125	0.57	0.12	0.037	0.275	0.09	0.09	0.105
I_min_	A	4	3	12	27	4	10	26	18
k_min_		0.121	0.242	0.078	0.034	0.137	0.082	0.073	0.065
I_sr_	A	5.375	5.625	16	27.87	6.125	10.875	30.625	26.25
k_sr_		0.158	0.302	0.087	0.036	0.18	0.082	0.082	0.072

**Table 4:** Experimental data of electric current variations.

Measurements							
1	2	3	4	5	6	7	8
I [A]							
1	1	4	0	1	0	2	2
1	1	5	0	1	0	2	2
1	1	12	0	1	0	2	2
1	1	12	0	1	0	1	3
2	1	12	0	1	0	1	4
4	3	12	0	4	0	18	4
4	3	16	4	4	3	18	4
5	3	16	4	5	3	32	4
4	6	17	5	5	3	32	18
4	6	16	5	6	3	33	17
4	6	17	3	6	3	32	27
5	7	16	3	6	3	32	27
5	6	16	3	6	4	33	27
5	6	16	3	6	4	33	27
5	6	17	28	6	4	30	28
5	6	17	28	6	4	28	28
6	7	16	29	6	3	28	28
6	7	17	28	7	3	29	29
6	7	17	28	7	10	31	28
7	7	17	28	7	11	31	29
5	7	17	29	7	11	31	29
5	7	16	28	7	11	31	29
5	7	16	28	7	11	29	27
6	7	16	28	7	11	29	27
6	7	16	27	6	11	29	27
6	6	16	27	6	11	30	26
6	6	17	27	6	11	30	26
6	6	17	27	7	11	30	26
6	6	17	28	7	11	30	26
6	6	17	28	7	11	30	27
6	6	17	28	7	10	31	26
6	6	16	28	7	10	31	28
5	6	16	29	8	10	31	28
5	6	16	29	8	11	32	24
5	6	16	27	8	11	31	24
5	6	16	27	6	11	29	25
2	6	17	27	6	11	29	6
2	3	4	28	6	11	32	6
2	3	4	28	7	10	33	6
2	3	3	28	7	10	33	4
2	3	3	3	2	11	33	4
1	3	2	3	2	11	5	4
1	3	1	2	2	3	5	4
1	1	1	3	2	2	3	4
1	1	1	3	2	2	3	4
1	1	1	3	1	1	3	4

## Data Availability

Data is contained within the article.
